# *N*-[2-(4-Acetyl-1-Piperazinyl)Phenyl]-2-(3-Methylphenoxy)Acetamide (NAPMA) Inhibits Osteoclast Differentiation and Protects against Ovariectomy-Induced Osteoporosis

**DOI:** 10.3390/molecules25204855

**Published:** 2020-10-21

**Authors:** Jinkyung Lee, Sun-Hee Ahn, Zhihao Chen, Sohi Kang, Dong Kyu Choi, Changjong Moon, Sang Hyun Min, Byung-Ju Park, Tae-Hoon Lee

**Affiliations:** 1 Department of Oral Biochemistry, Dental Science Research Institute, School of Dentistry, Chonnam National University, Gwangju 61186, Korea; wlsrud1945@naver.com; 2 Department of Bio-Health Research Center, Korea Photonics Technology Institute (KOPTI), Gwangju 61007, Korea; sun3193@kopti.re.kr; 3 Department of Molecular Medicine (BK21plus), Chonnam National University Graduate School, Gwangju 61186, Korea; chinaczhihao@gmail.com; 4 Department of Veterinary Anatomy, College of Veterinary Medicine and BK21 FOUR Program, Chonnam National University, Gwangju 61186, Korea; shrloveu@gmail.com (S.K.); moonc@chonnam.ac.kr (C.M.); 5 New Drug Development Center, DGMIF, 80 Chumbok-ro, Dong-gu, Daegu 41061, Korea; dongkyu@dgmif.re.kr (D.K.C.); shmin03@dgmif.re.kr (S.H.M.)

**Keywords:** NAPMA, osteoclast, ovariectomy, osteoporosis, bone loss, bone resorption

## Abstract

Osteoclasts are large, multinucleated cells responsible for bone resorption and are induced in response to the regulatory activity of receptor activator of nuclear factor-kappa B ligand (RANKL). Excessive osteoclast activity causes pathological bone loss and destruction. Many studies have investigated molecules that specifically inhibit osteoclast activity by blocking RANKL signaling or bone resorption. In recent years, we screened compounds from commercial libraries to identify molecules capable of inhibiting RANKL-induced osteoclast differentiation. Consequently, we reported some compounds that are effective at attenuating osteoclast activity. In this study, we found that *N*-[2-(4-acetyl-1-piperazinyl)phenyl]-2-(3-methylphenoxy)acetamide (NAPMA) significantly inhibited the formation of multinucleated tartrate-resistant acid phosphatase (TRAP)-positive cells from bone marrow-derived macrophages in a dose-dependent manner, without cytotoxic effects. NAPMA downregulated the expression of osteoclast-specific markers, such as c-Fos, NFATc1, DC-STAMP, cathepsin K, and MMP-9, at the transcript and protein levels. Accordingly, bone resorption and actin ring formation were decreased in response to NAPMA treatment. Furthermore, we demonstrated the protective effect of NAPMA against ovariectomy-induced bone loss using micro-CT and histological analysis. Collectively, the results showed that NAPMA inhibited osteoclast differentiation and attenuated bone resorption. It is thus a potential drug candidate for the treatment of osteoporosis and other bone diseases associated with excessive bone resorption.

## 1. Introduction

Bone homeostasis is a dynamic process that is important to maintain the balance between bone resorption by osteoclasts and bone formation by osteoblasts [1]. An imbalance caused by excessive osteoclast-induced bone resorption may lead to bone-destructive conditions, such as rheumatoid arthritis, osteoporosis, and lytic bone metastases [2].

Osteoclasts, which originate from hematopoietic cells of monocyte or macrophage lineage, undergo differentiation to form multinucleated cells (MNCs), which are responsible for bone resorption [3]. As osteoclasts are the primary bone resorptive cells, excessive osteoclast differentiation is strongly correlated with various bone diseases in humans. For example, excessive osteoclast-induced bone resorption may cause osteoporosis, rheumatoid arthritis, and osteoarthritis, and osteoclast dysfunction may cause osteopetrosis [4,5,6]. Osteoporosis is a disease characterized by the loss of bone mass, structural deterioration of bone tissue, and porous bones, all of which result in increased bone fragility [7]. Given the dramatic increase in the global average life expectancy, osteoporosis has come to be recognized as a critical public health issue [8]. Therefore, there is a need to develop an effective treatment for osteoporosis based on the inhibition of excessive osteoclast activity.

Osteoclast differentiation is regulated by a variety of systemic hormones and cytokines. Among these, the receptor activator of nuclear factor-kappa Β ligand (RANKL) and macrophage colony-stimulating factor (M-CSF) are known to play essential roles in the early stage of osteoclast differentiation [9,10]. The binding of RANKL to the receptor activator of nuclear factor-kappa Β (RANK) leads to the recruitment of tumor necrosis factor receptor-associated factor 6 (TRAF6), which triggers the activation of mitogen-activated protein kinases (MAPKs), extracellular signal-regulated kinase (ERK), c-Jun N-terminus kinase (JNK), and p38, and transcription factors, such as NF-kB, activator protein 1 (AP-1), and nuclear factor of activated T-cells c1 (NFATc1) [11,12]. The activation of these transcription factors triggers osteoclast differentiation via the upregulation of osteoclast-associated genes, such as tartrate-resistant acid phosphatase (TRAP), cathepsin K (Cts K), matrix metalloproteinase 9 (MMP-9), and dendritic cell-specific transmembrane protein (DC-STAMP) [13,14].

Several compounds have been reported to inhibit excess bone resorption or enhance bone formation [15,16]. One such compound is rotenone, which suppresses RANKL-induced osteoclast differentiation by blocking the MAPK signaling pathways [17]. α-Lipoic acid has also been reported to suppress bone resorption in vivo by inhibiting osteoclast differentiation [18]. Moreover, several small molecules and antibodies (against estrogen receptor modulators or RANKL) suppress osteoclast-mediated bone resorption. Bisphosphonates are well-known anti-resorption agents that can effectively treat various bone loss-related diseases [19] and suppress osteoclast formation and activity by binding to hydroxyapatite [20]. However, there are concerns regarding the adverse effects of bisphosphonates [21].

In recent years, we reported several compounds that target osteoclast inhibition [22,23,24]. In the present study, we selected a new chemical with a structure similar to that of previously reported chemicals that exhibit strong inhibitory effects on osteoclast differentiation in vitro, as well as an effect in vivo. In this paper, we describe this chemical, *N*-[2-(4-acetyl-1-piperazinyl)phenyl]-2-(3-methylphenoxy)acetamide (NAPMA), which inhibited RANKL-induced osteoclastogenesis; we evaluated the in vitro effect of NAPMA on the formation of TRAP-positive multinucleated cells and the bone resorptive activity of osteoclasts. Furthermore, we investigated the in vivo protective effect of NAPMA against bone loss in a mouse model of ovariectomy-induced osteoporosis.

## 2. Results

### 2.1. NAPMA Inhibits RANKL-Induced Osteoclastogenesis In Vitro but Does Not Affect the Differentiation of Primary Calvarial Cells Into Osteoblasts

**Figure 1:**
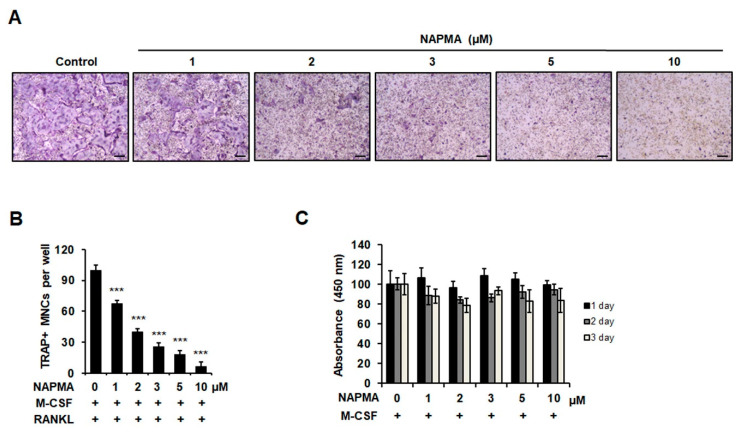
Effect of *N*-[2-(4-acetyl-1-piperazinyl)phenyl]-2-(3-methylphenoxy)acetamide (NAPMA) on receptor activator of nuclear factor-kappa B (RANKL)-induced osteoclast differentiation. (**A**) NAPMA inhibited osteoclast formation in a dose-dependent manner. Bone marrow-derived macrophages (BMMs; 1 × 10⁴ cells/well) were treated with different concentrations of NAPMA for three days in the presence of RANKL (50 ng/mL) and macrophage colony-stimulating factor (M-CSF; 30 ng/mL). The cells were fixed and stained for the tartrate-resistant acid phosphatase (TRAP) assay. (**B**) TRAP-positive multinucleated osteoclasts (≥3 nuclei) were counted. The data presented are the mean ± standard deviation (SD) of four independent experiments. Scale bar = 200 μm. *** *p* < 0.001 compared with the control group (treated M-CSF and RANKL but without NAPMA). (**C**) Effects of NAPMA on bone marrow-derived macrophages (BMM) viability after treatment for 72 h, as measured using the MTT assay. The data presented are the mean ± SD of three independent experiments.

We first examined the effect of various doses of NAPMA on RANKL-induced osteoclast formation. The formation of TRAP-positive osteoclasts decreased as the concentration of NAPMA increased (Figure 1A). When we quantified the number of TRAP-positive multinuclear cells (MNCs) in response to treatment with various concentrations of NAPMA, we observed that 30% and 50% of TRAP-positive MNC formation was suppressed by 1 and 2 μM NAPMA, respectively, compared with the control treatment (without NAPMA; Figure 1B). In particular, osteoclast differentiation was completely blocked by 10 μM NAPMA. To determine whether the suppressive effect of NAPMA on osteoclast formation was due to cytotoxic effects or the inhibition of proliferation, we determined the toxicity of NAPMA by measuring the viability and proliferation of bone marrow-derived macrophages (BMMs) treated with different concentrations of NAPMA using the 3-(4,5-dimethylthiazol-2-yl)-2,5-diphenyltetrazolium bromide (MTT) assay and BrdU Assay Kit, respectively. NAPMA did not exert a cytotoxic effect on BMMs (Figure 1C) or significantly inhibit the proliferation of BMMs (Supplemental Figure S1).

To investigate the stage of osteoclastogenesis that was affected by NAPMA, BMMs were treated with 5 or 10 μM NAPMA at different time points and incubated accordingly (Supplemental Figure S2). The results showed that treatment with NAPMA on the first day of differentiation and continued application during the differentiation process resulted in the potent inhibition of RANKL-induced osteoclast differentiation. Moreover, following NAPMA treatment on later days (day 2 or 3) of differentiation, much higher levels of osteoclast differentiation occurred than after NAPMA treatment on the first day of differentiation. This suppressive effect was directly related to the concentration of NAPMA. Interestingly, we observed that NAPMA treatment on day 3 of differentiation still resulted in 50% inhibition of osteoclast differentiation compared with the control treatment (without NAPMA). These findings indicated that the inhibition of RANKL-induced osteoclastogenesis by NAPMA occurred not only at the beginning of differentiation, but also during the later stages of differentiation. This suggested that NAPMA influences the pathways that are important for both the early and late stages of osteoclast formation.

Furthermore, to examine whether NAPMA affected osteoblastogenesis in vitro, we treated BMP-induced differentiated osteoblasts with different concentrations of NAPMA (2 to 10 μM) and examined ALP staining and activity. In addition, we analyzed the expression of osteoblast-specific markers, such as ALP, RANKL, and osteocalcin (OCN). As shown in Supplemental Figure S3, NAPMA treatment did not influence the expression of osteoblast differentiation (Supplemental Figure S3A), ALP activity (Supplemental Figure S3B), and differentiation markers ALP, RANKL, and osteocalcin (Supplemental Figure S3C) [25].

### 2.2. NAPMA Suppresses the Expression of Osteoclast Marker Genes

**Figure 2:**
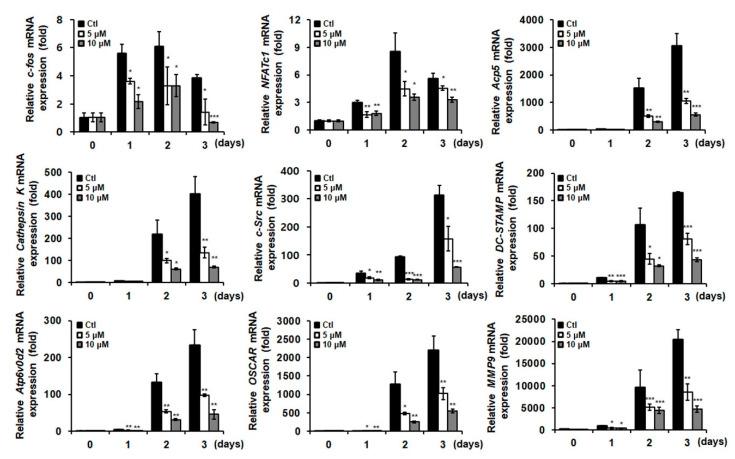
NAPMA inhibits the expression of osteoclast-specific genes. qPCR analysis of the expression of osteoclast-specific genes, *c-Fos, NFATc1, Acp5, cathepsin K, c-Src, DC-STAMP, Atp6v0d2, OSCAR,* and *MMP9* in BMMs stimulated with RANKL for three days in the presence of NAPMA. The data presented are the mean ± SD of three independent experiments. * *p* < 0.05, ** *p* < 0.01, and *** *p* < 0.001 compared with the control group (treated RANKL but without NAPMA).

To further elucidate the role of NAPMA in osteoclast differentiation, we analyzed the expression of osteoclast marker genes during RANKL-induced osteoclastogenesis using quantitative PCR. The expression of several osteoclast-specific genes, including *c-Fos*, *NFATc1*, *Acp5*, *cathepsin K*, *c-Src*, *DC-STAMP*, *Atp6v-d2*, *OSCAR*, and *MMP9*, was upregulated in BMMs when osteoclast differentiation was induced, but was suppressed in a dose-dependent manner in response to NAPMA treatment (Figure 2). *c-Fos* and *NFATc1* are critical for early osteoclast differentiation [26]: *c-Fos* is recruited to the *NFATc1* promoter during the early stages of osteoclast differentiation [27]. We also observed that the expression of *c-Fos* and *NFATc1* was upregulated on day 1 of differentiation, unlike other marker genes, which began to be increased on day 2 of differentiation. Moreover, the inhibitory effect of NAPMA on osteoclast differentiation was consistently observed in RAW264.7 cells in a dose-dependent manner (Supplemental Figure S4).

**Figure 3:**
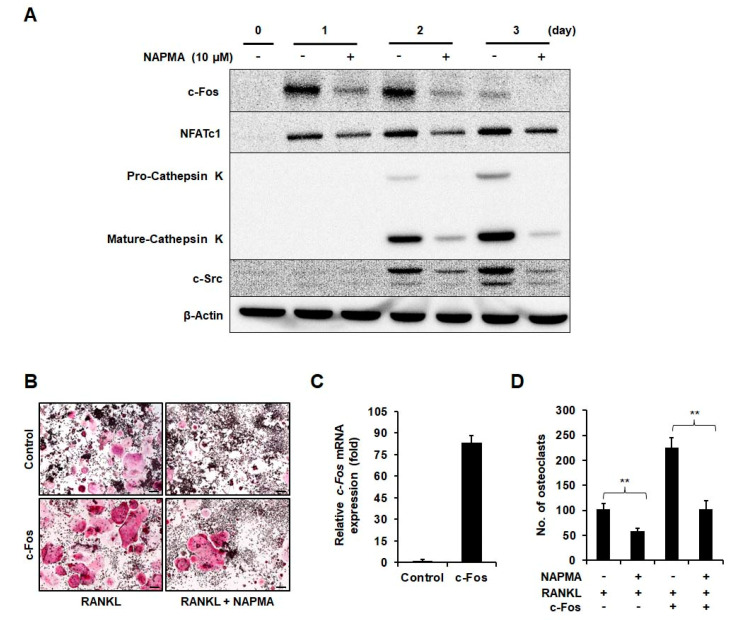
NAPMA suppresses the expression of c-Fos, NFATc1, cathepsin K, and c-Src. (**A**) NAPMA inhibits the RANKL-induced expression of c-Fos, NFATc1, cathepsin K, and c-Src proteins. BMMs were treated with or without NAPMA (10 μM) in the presence of RANKL (50 ng/mL) and M-CSF (30 ng/mL). Protein expression was examined using western blotting after the indicated treatment times. (**B**) RAW264.7 cells were transfected with empty vector (Control) or c-Fos vector (c-Fos) and then incubated with or without NAPMA (10 μM) in the presence of RANKL (50 ng/mL). The data presented are the mean ± SD of four independent experiments. Scale bar = 200 μm. After three days, the cell was fixed and stained for the TRAP assay. (**C**) The expression of c-Fos was analyzed by real-time PCR in empty vector- and c-Fos vector-transfected RAW264.7 cells. (**D**) TRAP-positive multinucleated osteoclasts (≥3 nuclei) were counted. The data are presented as the mean ± SD of three independent experiments. ** *p* < 0.01.

Consequently, we observed a significant downregulation of c-Fos, NFATc1, and cathepsin K protein expression in osteoclast cells in response to NAPMA treatment (Figure 3A). Interestingly, the expression of c-Src protein, which is known to be important for actin dynamics and its organization in osteoclasts [28], was downregulated in NAPMA-treated osteoclasts. Next, we hypothesized that NAPMA specifically acts via the inhibition of c-Fos, which is the key factor in the early stages of osteoclast differentiation. To support this hypothesis, we tested whether NAPMA still inhibited the differentiation of c-Fos-overexpressing RAW264.7 cells to osteoclasts. The results showed that the overexpression of c-Fos dramatically increased RANKL-induced osteoclastic differentiation in RAW264.7 cells in the absence of NAPMA (no. of osteoclasts: 102 ± 11 for empty vector vs. 225 ± 19 for c-Fos-overexpressing cells). In RAW264.7 cells overexpressing c-Fos, NAPMA treatment significantly decreased osteoclastic differentiation (no. of osteoclasts: 225 ± 19 without NAPMA vs. 103 ± 16 with NAPMA) (Figure 3B–D).

Furthermore, we examined the impact of NAPMA on the MAPK, NF-kB, and NFATc1 pathways. The phosphorylation of three MAPK family members (ERK, JNK, and p-38) and NF-kB was upregulated in response to RANKL stimulation [29]. We found that NAPMA treatment did not significantly affect the phosphorylation of JNK, p-38, ERK, NF-kB, IkB, and Akt (Supplemental Figure S5) or the expression of TRAF6, calcineurin A and B, and calmodulin proteins (Supplemental Figure S6A). Moreover, we examined whether NAPMA was involved in the PLCγ-Ca^2+^-calcineurin pathway of NFATc1 activation and found that NAPMA had no effect on the phosphorylation of PLCγ and calcineurin phosphatase activity (Supplemental Figure S6B,C).

### 2.3. NAPMA Inhibits Bone Resorption and Actin Ring Formation in Osteoclasts

**Figure 4:**
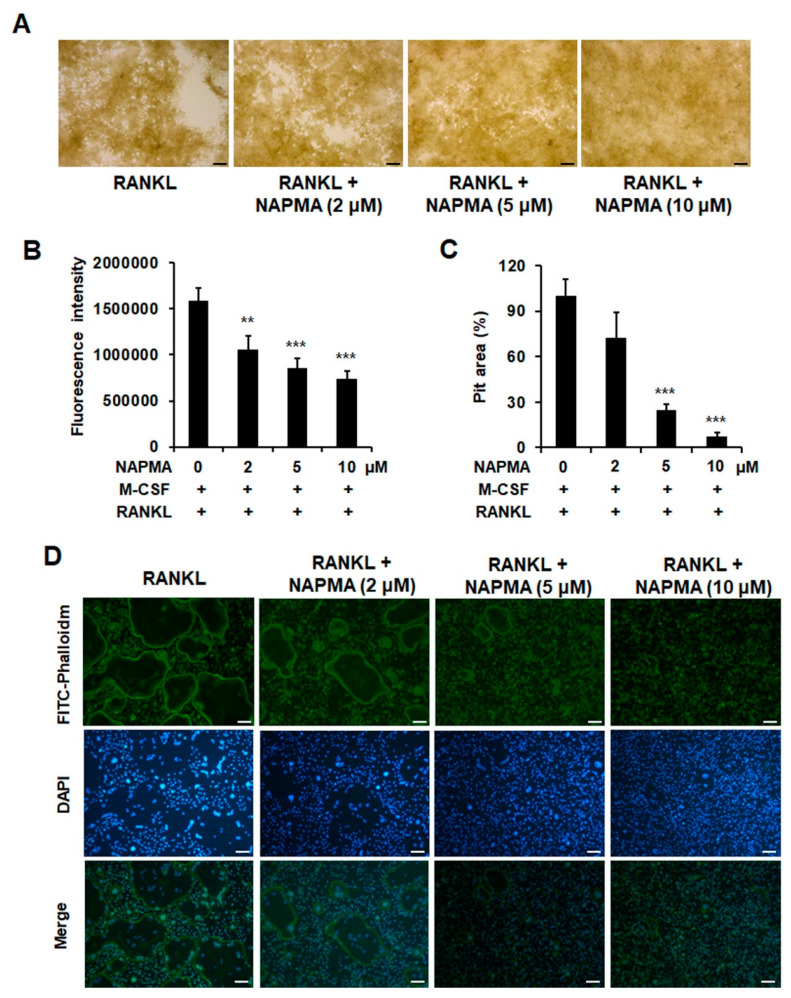
NAPMA inhibits osteoclast formation. (**A**) NAPMA inhibits bone resorption of mouse osteoclasts on bovine bone slices treated with dimethyl sulfoxide (DMSO) (control) or various concentrations of NAPMA for six days. Scale bar = 200 μm. (**B**) Fluorescence intensity was measured at an excitation wavelength of 485 nm and an emission wavelength of 535 nm on a fluorometric plate reader. (**C**) The area of the resorption pits was measured. The data presented are the mean ± SD of three independent experiments. ** *p* < 0.01; *** *p* < 0.001. (**D**) NAPMA disrupts actin ring formation by mature osteoclasts. After culturing for 72 h, staining for actin ring formation was performed, followed by examination via fluorescence microscopy. Scale bar = 200 μm.

The formation of filamentous-actin (F-actin) ring structures in osteoclasts is a critical indicator of the bone resorption activity of osteoclasts. We, therefore, examined whether NAPMA inhibited osteoclast function by measuring bone resorption and actin ring formation. Assay plates were coated with fluoresceinamine-labeled chondroitin sulfate (FACS) and calcium phosphate (CaP) [30]. Then, bone marrow cells were seeded in the presence of 30 ng/mL M-CSF and 50 ng/mL RANKL, with or without NAPMA (2, 5, and 10 μM). As expected, NAPMA caused a significant decrease in bone resorption, and the suppressive effect of NPMA was dependent on its concentration (Figure 4A).

The coated calcium phosphate was first bound to fluoresceinamine-labeled chondroitin sulfate (FACS), which was released from the calcium phosphate layer into the medium by osteoclastic resorption activity. Bone resorption activity was evaluated through the measurement of the fluorescence intensity of the medium. We observed a reduction in bone resorption upon measuring fluorescence intensity (Figure 4B) and in the pit area (Figure 4C) and found that the suppressive effect was dependent on the NAPMA concentration. Consequently, we showed that NAPMA treatment disrupted F-actin ring formation in a dose-dependent manner (Figure 4D).

### 2.4. NAPMA Inhibits Osteoclast Differentiation to a Greater Extent than PPOA Derivatives

We previously reported that PPOA-N-Ac-2-Me and PPOA-N-Ac-2-Cl exert inhibitory effects on osteoclast formation [24]. Owing to their structural similarity to NAPMA, we examined whether the inhibitory effect of NAPMA on RANKL-induced osteoclast differentiation was comparable with that of PPOA-N-Ac-2-Me or PPOA-N-Ac-2-Cl. Interestingly, NAPMA and PPOA-N-Ac-2-Cl showed the strongest inhibitory effect on osteoclast differentiation (Supplemental Figure S7). We also observed that PPOA-N-Ac-2-Me was less effective in inhibiting osteoclast differentiation than PPOA-N-Ac-2-Cl, as expected from the previous report [24].

Based on these inhibitory effects, we tested the in vivo effects of NAPMA and PPOA-N-Ac-2-Cl (at the same dose) on ovariectomized (OVX)-induced osteoporosis in mice using a Quantum GX Micro-CT imaging system. As shown in Supplemental Figure S8, micro-CT analysis revealed that NAPMA treatment restored the OVX-associated bone loss, whereas PPOA-N-Ac-2-Cl did not affect bone loss (Supplemental Figure S8A). In addition, compared with PPOA-N-Ac-2-Cl treatment, NAPMA treatment for six weeks significantly increased the bone mineral density (BMD) of the femur (Supplemental Figure S8B).

### 2.5. NAPMA Attenuates OVX-Induced Bone Loss in Mice

**Figure 5:**
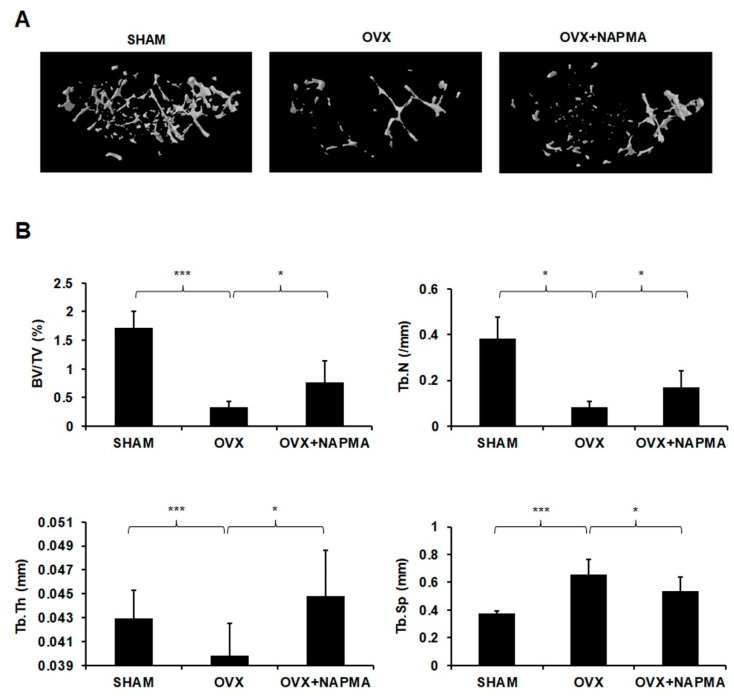
NAPMA inhibits OVX-induced bone loss. (**A**) OVX mice were euthanized after six weeks of NAPMA treatment. Micro-CT images of the distal femurs from the sham-operated group (SHAM), OVX group, and OVX + NAPMA group (NAPMA 10 mg/kg/each day) were obtained. (**B**) Quantitative analyses of BV/TV, Tb.N, Tb.Th, and Tb.Sp (N = 6 per group). * *p* < 0.05; *** *p* < 0.001.

We then assessed the effects of NAPMA on ovariectomized (OVX)-induced osteoporosis in mice using a micro-computed tomography (micro-CT) system (Skyscan 1172, Kontich, Belgium). The mice were acclimatized to the laboratory environment for one week and then underwent either a sham operation (n = 6) or an ovariectomy (n = 12). In the OVX group, both ovaries were removed from each animal. In the sham-operated group, to exert the same environmental stresses, the animals underwent a laparotomy, but the ovaries were not removed. The OVX mice showed marked atrophy and decreased the wet weight of the uterus compared with the sham-operated mice (Supplemental Figure S9). Micro-CT showed that OVX induced significant bone loss, but treatment with NAPMA restored the OVX-associated bone loss (Figure 5A). Moreover, NAPMA prevented the OVX-induced reduction in trabecular bone volume (BV/TV), trabecular number (Tb.N), and trabecular thickness (Tb.Th) and the OVX-induced increase in trabecular separation (Tb.Sp; Figure 5B), suggesting that NAPMA significantly inhibited OVX-induced bone loss.

**Figure 6:**
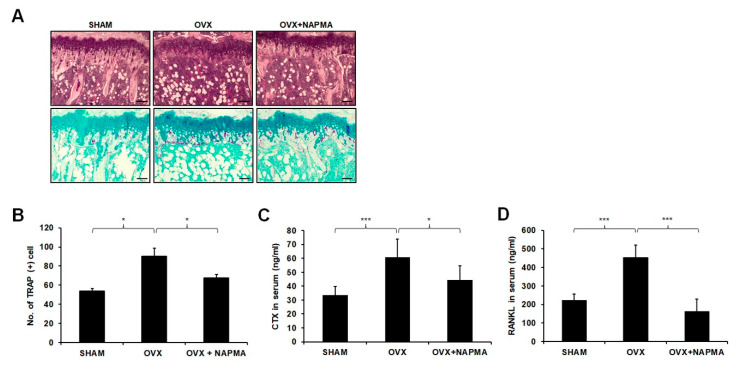
(**A**) Representative hematoxylin and eosin (H&E) and TRAP staining of decalcified femur sections from sham, OVX, and OVX + NAPMA mice from each group at six weeks after the operation. Scale bar = 200 μm. (**B**) The number of Trap-positive cells was quantified via TRAP-staining. Serum levels of (**C**) CTX-1 and (**D**) RANKL in mice treated with NAPMA. Mice received a sham operation or ovariectomy as described and were treated with a vehicle or NAPMA (10 mg/kg/each day) for six weeks. The serum was collected before the animals were euthanized. The data are presented as the mean ± SD. N = 6. * *p* < 0.05; *** *p* < 0.001.

Consistent with the micro-CT data, histological analysis showed significant reductions in trabecular bone parameters in the OVX-induced mice, which were restored upon NAPMA treatment (Figure 6A). Accordingly, the number of TRAP-positive cells was reduced upon NAPMA treatment (Figure 6B). Moreover, the serum concentration of type 1 collagen cross-linked C-terminal telopeptide (CTX-1), a bone resorption marker, was increased in the OVX mice compared with the sham-operated control mice, whereas NAPMA treatment significantly decreased the OVX-induced CTX-1 levels (Figure 6C). As the balance between RANKL and osteoprotegerin (OPG) produced by osteoblast lineage cells is critical for osteoclastogenesis and function, we examined the serum levels of RANKL and OPG using ELISA. In the OVX mice, serum levels of RANKL were increased, whereas NAPMA treatment clearly decreased the OVX-induced RANKL levels (Figure 6D). The serum OPG levels in the OVX mice were not significantly different from those in the sham-operated mice and NAPMA-treated mice (Supplemental Figure S10A). Hence, the RANKL/OPG ratio was significantly lower in OVX mice treated with NAPMA compared with that in the untreated OVX mice (Supplemental Figure S10B). Moreover, the serum level of the inflammatory cytokine IL-6 dramatically increased in the OVX mice compared with that in the SHAM mice, although NAPMA treatment decreased the OVX-induced IL-6 level (Supplemental Figure S10C).

## 3. Discussion

Bone homeostasis is a tightly regulated process that is associated with the balance between osteoblast and osteoclast activity; together, these activities maintain bone mass and mineral homeostasis [31]. During bone regeneration, these functions are controlled by the bone microenvironment and the cellular signal transduction systems that regulate osteoblast and osteoclast differentiation [32]. Excessive osteoclast activity is observed in many osteoclast-related diseases, including osteoporosis, periodontitis, and rheumatoid arthritis [33]. Osteoporosis is a serious disease that is common in postmenopausal women and older people in developed countries [34]. In the elderly population, the balance between bone destruction-bone resorption plays a very important role in the condition of patients with osteoporosis and is considered a therapeutic goal [35,36]. Many reports have suggested that several compounds ameliorate bone loss in osteoporosis through the inhibition of the signaling pathways involved in osteoclast differentiation [37,38,39]. In the present study, we investigated the effects of NAPMA on RANKL-induced osteoclastogenesis in vitro and on OVX-induced bone loss in vivo. NAPMA treatment reduced osteoclast formation compared with that observed with RANKL stimulation in a dose-dependent manner, and this effect was not due to the cytotoxicity of NAPMA. However, NAPMA did not affect the osteoblast differentiation of primary calvarial cells.

The interaction of RANKL with its receptor, RANK, triggers the activation of various signaling cascades during osteoclast differentiation. Signaling pathways, including MAPKs, NF-kB, and activator protein-1 (AP-1), are essential for osteoclast differentiation [40]. NAPMA did not affect the activation of MAPKs or NF-kB in response to RANKL stimulation (Figure S4). This phenomenon occurred in the presence, as well as the absence, of M-CSF during RANKL-induced signaling (data not shown). Therefore, it was unlikely that NAPMA directly regulated the activation of signaling downstream of MAPKs/NF-kB. Nevertheless, NAPMA treatment reduced NFATc1 expression in a dose-dependent manner, which subsequently affected the expression of typical osteoclast markers.

Collectively, our data showed that NAPMA inhibits the expression of c-Fos and NFATc1. Interestingly, chemical compound PPOA derivatives, which have a structure similar to that of NAPMA, have been reported to inhibit the RANKL-induced osteoclast formation of the NF-kB and MAPK subfamily, including p38, JNK, and ERK. Although the inhibitory effects of NAPMA and PPOA on osteoclast differentiation were similar in vitro, the point of action in the signaling pathway was different. This might lead to different inhibitory effects against osteoporosis in vivo.

The transcription factor NFATc1 is a well-known master regulator of RANKL-induced osteoclast differentiation [41]. NAFTc1 plays a critical role in osteoclast fusion and osteoclast activation via the upregulation of various genes responsible for osteoclast adhesion and migration and the acidification and degradation of the bone matrix [42]. In addition, c-Fos, which is known for its critical role in regulating osteoclast differentiation genes [43], and NFATc1 expression are induced through various signaling pathways via RANKL [17]. Arai et al. demonstrated that c-Fos knockout mice exhibited osteopetrosis, due to osteoclast deficiency [43]. Moreover, NFATc1 has been shown to restore osteoclastogenesis in cells lacking c-Fos. It is well established that c-Fos is essential for the RANKL-mediated induction of NFATc1 [41] and that these two transcription factors are functionally linked [43]. After RANKL triggers osteoclast differentiation, c-Fos is expressed in the early stages of osteoclast differentiation, where it further regulates NFATc1 gene expression by binding to the promoter region of NFATc1 [12]. In this study, we found that NAPMA downregulated the transcript and protein levels of c-Fos and NFATc1 at the beginning of differentiation. However, it did not influence the MAPKs, NF-kB, and calcineurin signaling pathways. Nevertheless, NAPMA negatively regulated the expression of osteoclast markers, including *Acp5*, *cathepsin K*, *MMP9*, *c-Src*, *OSCAR*, and *Atp6v0d2* (Figure 2 and Figure 3, Supplemental Figures S5 and S6). Similarly, Choi et al. also reported that the CGE extract did not inhibit the MAPK and NF-kB signaling pathways, but suppressed RANKL-induced c-Fos and NFATc1. It also affected the expression of osteoclast-related genes, such as *OSCAR*, *MMP9*, and *cathepsin K* [44].

Actin cytoskeletal organization includes the ruffled membrane, and the actin ring or sealing zone is essential to allow mature osteoclasts to perform bone resorption. Mice deficient in the *c-Src, Syk*, and *PLCγ2* genes exhibit impaired actin cytoskeletal organization in osteoclasts [45]. In this study, RANKL stimulated c-Src; NAPMA treatment decreased the expression of the c-Src protein, a well-known marker of late osteoclastogenesis associated with bone resorption (Figure 3), and showed an inhibitory effect on the RANKL-mediated actin ring formation of mature Ocs (Figure 4), indicating that the effect of NAPMA on c-Src activity may influence regulating RANKL-stimulated actin ring formation.

Despite the suppressive effect of NAPMA on osteoclastogenesis and bone resorption, it did not affect the osteoblast differentiation associated with bone formation. Similarly, Chen et al. also reported that treatment with the chemical IMD 0354 inhibited osteoclastogenesis without affecting bone formation [46]. They showed the suppressive effect of IMD 0354 on the expression of NFATc1 and c-Fos in vitro and further demonstrated the preventive effect of IMD 0354 on bone loss in vivo.

In our in vivo model, the serum level of CTX-1 was decreased upon NAPMA treatment in the OVX mice as expected from the micro-CT and histological data. Interestingly, the serum levels of RANKL and IL-6 also declined in the OVX mice treated with NAPMA. Our findings are consistent with some other reports. For example, Wang et al. [47,48] reported that phillyrin showed a protective effect against bone loss in an in vivo model by inhibiting RANKL-induced osteoclast differentiation, but did not affect osteoblast differentiation. Interestingly, phillyrin decreased RANKL expression in an in vivo model of osteolysis. Moreover, Zhong et al. [49] reported that tetrandrine inhibited osteoclast differentiation in bone marrow monocytes (BMMs) and RAW264.7 cells, but had no effect on osteoblast differentiation in vitro. Notably, tetrandrine prevented bone loss in OVX mice, resulting in a lower level of RANKL and cytokines in the serum, which was consistent with our observations. Collectively, these results show that NAPMA may also influence RANKL expression in osteoblasts; however, this requires further investigation.

In this study, we found similar in vitro inhibitory effects for NAPMA and PPOA-N-Ac-2-Cl (data previously reported). However, of these two compounds, only NAPMA showed a protective effect against bone loss in OVX-induced mice, as shown in the analysis of BMD. BMD is an important factor in determining bone strength, and there is strong evidence that the structure of cancellous bone plays a significant role in bone strength and determines its biomechanical properties [50].

Furthermore, we demonstrated that NAPMA exhibited a remarkable protective effect against OVX-induced bone loss in a mouse model, as confirmed by micro-CT (Figure 5), hematoxylin and eosin (H&E), and TRAP staining (Figure 6A).

To observe the lesions and structure of the cancellous bone, we further explained the change in BMD using a micro-CT voxel-based test unit scan. The micro-CT scan results showed that NAPMA increased BV/TV, Tb.Th, and Tb.N and decreased Tb.Sp compared with those in the OVX group. Furthermore, whether the administration of a clinical dosage of NAPMA can exert anti-osteoporotic effects in humans requires further investigation, as do the potential adverse effects in vivo.

In summary, to the best of our knowledge, this is the first study to show that NAPMA can inhibit osteoclast formation and function via the inhibition of c-Fos/NFATc1, which subsequently attenuates downstream osteoclast gene expression. In addition, NAPMA was found to prevent OVX-induced osteoporosis in vivo. Together, these results suggest that NAPMA is a potential therapeutic agent for osteoclast-related diseases, such as osteoporosis.

## 4. Materials and Methods

### 4.1. Ethics Statement

Mice were housed in a specific pathogen-free facility following the guidelines provided in the Guide for the Care and Use of Laboratory Animals (Chonnam National University, Gwangju, Korea). Adult female C57BL/6J mice (8-week-old) were used in this study. The collection of primary mononuclear cells from mice was approved by the IACUC at Chonnam National University (Approval No. CNU IACUC-YB-2017-70, 31 October 2017).

### 4.2. Reagents

*N*-[2-(4-acetyl-1-piperazinyl)phenyl]-2-(3-methylphenoxy)acetamide (NAPMA) was purchased from Hit2lead, San Diego, CA, USA; the compound is part of a commercially available compound library.

### 4.3. In Vitro Osteoclastogenesis Assay

For osteoclast differentiation in vitro, mouse bone marrow cells were isolated from the femurs and tibiae of 8-week-old C57BL/6J mice by flushing the bone marrow with α-minimum essential medium (MEM). The flushed cells were incubated in a culture medium (α-MEM containing 10% characterized heat-inactivated fetal bovine serum (chFBS) and 1% penicillin/streptomycin) for one day. To obtain primary BMMs, the suspended cells were then collected and cultured in a medium supplemented with 30 ng/mL M-CSF (PeproTech, Rocky Hill, NJ, USA) for three days. Thereafter, non-adherent cells were discarded, and the adherent cells (osteoclast precursors) were cultured in a medium supplemented with 30 ng/mL M-CSF and 50 ng/mL RANKL (PeproTech, Rocky Hill, NJ, USA) for up to three days; fresh medium was supplied every day. RAW264.7 cells were cultured for four days in a culture medium containing 50 ng/mL RANKL with or without NAPMA for osteoclastogenesis. To assess the extent of differentiation, the differentiated BMMs and RAW264.7 cells were fixed for 30 min in 3.7% formaldehyde prepared in phosphate-buffered saline and stained using a TRAP kit (Sigma-Aldrich, St. Louis, MO, USA). TRAP-positive multinucleate cells (containing five or more nuclei) were counted. After four days, the mature osteoclasts were counted under a microscope (DFC450 C, Leica Microsystems Ltd., Wetzlar, Germany) based on the number of nuclei (n ≥ 3). Each osteoclast formation assay was independently performed a minimum of three times.

### 4.4. Cell Viability Assay

To evaluate cytotoxicity, we used the MTT assay to measure cell viability following NAPMA treatment, as described previously [51,52]. Briefly, mouse bone marrow cells were prepared as described above, in Section 4.3. Osteoclast precursors were treated with 30 ng/mL M-CSF (PeproTech, Rocky Hill, NJ, USA) in a 96-well plate, in the presence or absence of NAPMA for three days; the MTT labeling reagent (0.5 mg/mL final concentration) was then added to each well. The cells were incubated for 4 h, followed by the addition of 100 μL 10% SDS in 0.01 M hydrochloric acid solution, and incubated for a further 3 h. The absorbance was measured at 450 nm using a microplate reader (Molecular Devices, San Jose, CA, USA—model: SpectraMax i3x).

### 4.5. Osteoclast Precursor Proliferation Assay

The proliferation of osteoclast precursor cells was quantified using a BrdU Cell Proliferation Assay Kit (GE Healthcare Life Sciences, Piscataway, NJ, USA) [53]. BMMs obtained from mice were treated with 30 ng/mL M-CSF (PeproTech, Rocky Hill, NJ, USA) for three days in the presence or absence of NAPMA at 37 °C in an atmosphere of 5% CO_2_ in the air. Cell proliferation was quantified based on BrdU incorporation using the BrdU Cell Proliferation ELISA Kit (Cell Signaling Technology, Boston, MA, USA).

### 4.6. In Vitro Osteoblastogenesis Assay

Primary mouse osteoblasts were isolated from the calvaria of 3-day-old C57BL/6J mice by sequential digestion with collagenase (Sigma-Aldrich, St. Louis, MO, USA), as previously described [54]. Primary osteoblasts were cultured in α-MEM supplemented with 10% characterized heat-inactivated FBS and 1% penicillin/streptomycin. After three days, the cells were reseeded (4 × 10^3^ cells/well) and cultured in an osteogenic medium containing 100 ng/mL bone morphogenic protein 2 (BMP-2) (Sino Biological, Wayne, PA, USA) with or without NAPMA (2, 5, and 10 μM, and control, respectively). The medium was exchanged for fresh medium every two days. After seven days of osteoblast differentiation, alkaline phosphatase (ALP) staining was performed, and ALP activity was assayed. For ALP staining, the cells were fixed with 70% ethanol for 1 h at room temperature and stained for 10 min with ALP staining solution following the manufacturer’s instructions (Sigma-Aldrich, St. Louis, MO, USA) [55]. ALP activity in the cells was measured using a TRAcP and ALP Assay Kit (TaKaRa Biotechnology, Otsu, Japan) following the manufacturer’s instructions. Each osteoblast differentiation assay was independently performed at least three times.

### 4.7. Plasmid Transfection

RAW264.7 cells were then transfected with pIRES-hrGFP-2a (empty vector) or pIRES-hrGFP-c-fos-2a (c-Fos vector) plasmid and Lipofectamine™LTX Reagent with PLUS™ Reagent (Thermo Fisher Scientific, Waltham, MD, USA) in a culture medium for 24 h; they were then seeded in a 6-well plate and treated with RANKL (50 ng/mL) and NAPMA (10 μM) for three days.

### 4.8. Real-Time PCR

Total RNA was isolated from BMMs treated with M-CSF and RANKL using the QIAzol RNA Lysis reagent (Qiagen Sciences, Valencia, CA, USA). cDNA was then synthesized using the PrimeScript™ RT Reagent Kit (Takara Biotechnology, Tokyo, Japan) following the manufacturer’s instructions. Quantitative PCR was performed using a QuantStudio 3 real-time PCR system (Applied Biosystems, Foster City, CA, USA) with a Power SYBR Green PCR Master Mix (Applied Biosystems, Foster City, CA, USA) and a standard temperature protocol. The results for the cycle threshold were expressed as relative quantities and calculated using the 2^−ΔΔ^CT method (expressed as the relative fold ratio). Glyceraldehyde 3-phosphate dehydrogenase (GAPDH) was used as the control gene for the normalization of mRNA expression. Three separate experiments were performed. The primers used for the quantitative real-time PCR assay are presented in Table 1.

### 4.9. Western Blotting

Osteoclasts were lysed in chilled lysis buffer [50 mM Tris–HCl (pH 7.5), 150 mM NaCl, 1% NP-40, 0.5% sodium deoxycholate, 0.1% sodium dodecyl sulfate (SDS), 2 mM EDTA, and protease inhibitors (Sigma-Aldrich, St. Louis, MO, USA)], and the supernatant was collected following centrifugation (10,000× *g*, 4 °C, 30 min). The concentration of proteins extracted from the differentiated osteoclasts was determined using the BCA protein assay (Pierce, Rockford, IL, USA). Proteins (20 μg) were separated on a 12% polyacrylamide gel electrophoresis (PAGE) gel and transferred onto polyvinylidene fluoride membranes (Bio-Rad Laboratories, Hercules, CA, USA). The membranes were then incubated with anti-β-actin (Sigma-Aldrich, St Louis, MO, USA), anti-NFATc1 (Cell Signaling Technology, Boston, MA, USA), anti-c-Fos (Cell Signaling Technology, Boston, MA, USA), anti-cathepsin K (Santa Cruz Biotechnology, Dallas, TX, USA), anti-TRAF6 (Santa Cruz Biotechnology, Dallas, TX, USA), anti-calcineurin A (Abcam, Cambridge, MA, USA), anti-Calcineurin B (Abcam, Cambridge, MA, USA), anti-calmodulin (Cell Signaling Technology, Boston, MA, USA), anti-c-Src (Cell Signaling Technology, Boston, MA, USA), anti-ERK1/2 (Cell Signaling Technology, Boston, MA, USA), anti-phospho-ERK1/2 (Cell Signaling Technology, Boston, MA, USA), anti-p38 (Cell Signaling Technology, Boston, MA, USA), anti-phospho-p38 (Cell Signaling Technology, Boston, MA, USA), anti-JNK (Cell Signaling Technology, Boston, MA, USA), anti-phospho-JNK (Cell Signaling Technology, Boston, MA, USA), anti-p65 (Cell Signaling Technology, Boston, MA, USA), anti-phospho-p65 (Cell Signaling Technology, Boston, MA, USA), anti-IkB (Cell Signaling Technology, Boston, MA, USA), anti-phospho-IkB (Cell Signaling Technology, Boston, MA, USA), anti-Akt (Cell Signaling Technology, Boston, MA, USA) or anti-phospho-Akt (Cell Signaling Technology, Boston, MA, USA), anti-PLCγ2 (Cell Signaling Technology, Boston, MA, USA), or anti-phospho-PLCγ2 (Cell Signaling Technology, Boston, MA, USA) antibodies. This was followed by probing with appropriate horseradish peroxidase (HRP)-conjugated secondary antibody (Cell Signaling Technology Boston, MA, USA). Chemiluminescence was detected using an ECL system (iNtRON, Seoul, Korea).

### 4.10. Resorption Pit Assay

For the bone resorption activity assay, the Bone Resorption Assay Kit (CosMo Bio, Tokyo, Japan) was used in accordance with the manufacturer’s instructions. BMMs were cultured on bone resorption assay plate 48 (2.5 × 10^4^ cells/well), in the presence of 2, 5, and 10 μM NAPMA or in the absence of NAPMA (control), along with M-CSF (30 ng/mL) and RANKL (50 ng/mL) for six days. On the seventh day after seeding, the culture supernatant was harvested into a 96-well black polypropylene microplate (Thermo Fisher Scientific Nunc, Waltham, MD, USA) and mixed with 0.1 N NaOH (50 μL). The fluorescence intensity was measured on a fluorescence plate reader (Molecular Devices, San Jose, CA, USA; model, SpectraMax i3x); the excitation and emission wavelengths were 485 nm and 535 nm, respectively. In addition, the pit areas were calculated by analyzing 10 randomly selected images (10× magnification) per well using ImageJ software (https://imagej.nih.gov/ij). The total resorption area was measured for each random picture, and the mean resorption area posteriorly calculated.

### 4.11. Actin Ring Formation Assay

The actin rings of the osteoclasts were detected by staining the actin filaments with Alexa Fluor™ 488 Phalloidin (Thermo Fisher Scientific, Waltham, MD, USA). Osteoclasts were generated from the culture of BMMs in the presence of M-CSF (30 ng/mL) and RANKL (50 ng/mL), with NAPMA (2, 5, and 10 μM) or without NAPMA (control). At the end of the incubation, osteoclasts were stained with Alexa Fluor™ 488 Phalloidin to observe actin. The distribution of the actin rings was visualized and detected using a fluorescence microscope (Carl Zeiss, Jena, Germany).

### 4.12. Calcineurin Activity Assay

The calcineurin activity was measured using the calcineurin cellular activity assay kit (Enzo Life science, Inc., Farmingdale, NY, USA) in accordance with the manufacturer’s instructions. Briefly, cultured RAW264.7 cells at semi-confluence (approximately 50%) were further cultured for 30 min with α-MEM, 10% FBS, and RANKL alone or in a medium containing NAPMA. Cell extracts from cultured cells were prepared with lysis buffer containing EDTA and EGTA (50 μM each) and centrifuged. Colorimetric measurements were performed at 620 nm.

### 4.13. In Vivo Animal Model

Eight-week-old female C57BL/6J mice were randomly divided into three groups (n = 6 mice per group): Sham-operated mice (SHAM), ovariectomized (OVX) mice treated with a vehicle (OVX), and OVX mice treated with NAPMA (OVX + NAPMA). As described previously, ovariectomy was performed by removing both the ovaries through a dorsal approach, and sham surgery was performed by identifying both ovaries. At one week after surgery, the mice were injected intraperitoneally (i.p.) with NAPMA (10 mg/kg) or vehicle each day for five weeks. After six weeks, all mice were euthanized, and the femurs and tibias were collected.

### 4.14. Micro-CT Imaging and Data Analysis

CT imaging was performed using a Quantum GX Micro-CT imaging system (PerkinElmer, Hopkinton, MA, USA), located at the Korea Basic Science Institute (Gwangju, Korea). The X-ray source was set to 90 kV and 88 mA with a field of view of 10 mm (voxel size, 20 μm; scanning time, 4 min). The 3D imaging was represented by 3D Viewer, in-built software within the Quantum GX, and the resolution was set at 4.5 μm to obtain images for visualization and display. Following scanning, the structural parameters for trabecular bone were analyzed using Analyze 12.0 software (AnalyzeDirect, Overland Park, KS, USA). The bone mineral density of the femur was estimated using a hydroxyapatite (HA) phantom (QRM-MicroCT-HA, Quality Assurance In Radiology and Medicine GmbH, Moehrendorf, Germany), scanned using the same parameters. The bone mineral density (BMD) of femurs was calculated using the ROI tool. Parameter values were shown as the mean ± standard deviation (SD).

### 4.15. Micro-CT

Bone morphometric parameters of femurs cleared of adherent soft tissues were assessed using a micro-CT system (Skyscan 1172, Kontich, Belgium). Scans were taken at a source voltage of 50 kV and a source current of 201 μA. The resolution was set at 10.7 μm, and the rotation step at 0°. 2D and 3D images were obtained for visualization and display. The structural parameters for trabecular bone were analyzed using CTAn software (Skyscan). Bone volume densities (BV/TV) and trabecular thickness/separation/number (Tb.Th, Tb.Sp, and Tb.N, respectively) values were calculated. The distal femur metaphysis was used as the region of interest for the analysis.

### 4.16. Tissue Preparation and Histomorphometric Analysis

Histological analysis was performed using a modified method for H&E and TRAP stains, as previously described. To prepare paraffin wax-embedded sections, the mice were sacrificed at six weeks (n = 6 mice/group). The isolated femur was fixed in 4% paraformaldehyde solution for 24 h and then immersed in 10% EDTA decalcifying solution for one week. After complete decalcification, paraffin was embedded and sectioned longitudinally (4-μm thickness). Before tartrate-resistant acid phosphatase (TRAP) staining, the sections were stained with H&E to detect structural changes. TRAP activity was demonstrated using an acid phosphate kit (Cat. No. PMC-AK04F-COS, Cosmo Bio Co., Tokyo, Japan). TRAP staining was performed in accordance with the manufacturer’s protocol. The sections were counter-stained with methyl green. TRAP-staining positive multinucleated cells containing at least three nuclei were counted as osteoclast cells. The number and area of TRAP-staining positive multinucleated cells were counted in a randomized order by the same researcher.

### 4.17. Measurement of Serum CTX-1, Osteocalcin, RANKL, OPG, IL-6 Levels

The serum was collected from the SHAM, OVX, and OVX + NAPMA mice before euthanasia after five weeks of NAPMA treatment. Serum CTX-1 levels were measured using a RatLaps EIA kit (IDS Nordic, Herlev, Denmark). Serum RANKL levels were measured using a Mouse RANKL ELISA kit (Abcam, Cambridge, MA, USA). Serum OPG levels were measured using a Quantikine ELISA (R&D system, Minneapolis, MN, USA). Serum interleukin-6 levels were measured using an ELISA MAX™ Deluxe Set Mouse IL-6 (BioLegend, San Diego, CA, USA).

### 4.18. Statistical Analysis

Statistical analyses were performed using unpaired two-tailed Student’s *t*-tests (* *p* < 0.05; ** *p* < 0.01; and *** *p* < 0.001; NS, not significant). All data are expressed as the mean ± SD, and the results represent more than three independent experiments.

## Figures and Tables

**Table 1:** Primers used in this study.

Gene	Primer Sequence (5′→3′)	
***GAPDH***	Forward	TGTGTCCGTCGTGGATCTGA
	Reverse	GATGCCTGCTTCACCACCTT
*c-Fos*	Forward	CGAAGGGAACGGAATAAGATG
	Reverse	GCTGCCAAAATAAACTCCAG
*NFATc1*	Forward	ACCACCTTTCCGCAACCA
	Reverse	GGTACTGGCTTCTCTTCCGTTTC
*Acp5*	Forward	CAGCTGTCCTGGCTCAAAA
	Reverse	ACATAGCCCACACCGTTCTC
*Cathepsin K*	Forward	GGACGCAGCGATGCTAACTAA
	Reverse	CAGAGAGAAGGGAAGTAGAGTTGTCACT
*c-Src*	Forward	CCAGGCTGAGGAGTGGTACT
	Reverse	CAGCTTGCGGATCTTGTAGT
*DC-STAMP*	Forward	CGCACGATGCTTCATTCTTC
	Reverse	CAGTGCCAGCCGCAATC
*ATP6v0d2*	Forward	GTGAGACCTTGGAAGACCTGAAA
	Reverse	TCCTCATCTCCGTGTCAATTTTG
*OSCAR*	Forward	TGGCGGTTTGCACTCTTCA
	Reverse	GGAAGAACTCAGCCAGCTCAA
*MMP9*	Forward	CTGGACAGCCAGACACTAAAG
	Reverse	CTCGCGGCAAGTCTTCAGAG

## Supplementary Materials

The following are available online, Figure S1: NAPMA had no effect on the proliferation of BMMs, Figure S2: Effects of NAPMA on RANKL-induced signaling in BMMs, Figure S3: NAPMA had no effect on BMP-induced osteoblast differentiation, Figure S4: Effect of NAPMA on receptor activator of nuclear factor-kappa B (RANKL)-induced osteoclast differentiation in RAW264.7 cells, Figure S5: Effects of NAPMA on RANKL-induced signaling in BMMs, Figure S6: NAPMA does not inhibit the RANKL-induced expression of (A) TRAF6, calmodulin, calcineurin A or B proteins, and (B) anti-phospho PLCγ, Figure S7: Effect of NAPMA, PPOA-N-Ac-2-Me, and PPOA-N-Ac-2-Cl on on RANKL-induced signaling in BMMs, Figure S8: NAPMA treatment prevented OVX-induced bone loss, Figure S9: Validation of the success of ovariectomy, Figure S10: The serum level of OPG (A), the RANKL:OPG ratio (B), and IL-6 (C) of the animals was determined using ELISA, Table S1: Primers used in this study.

## Abbreviations

RANKL	receptor activator of nuclear factor-kappa B ligand
NAPMA	N-[2-(4-acetyl-1-piperazinyl)phenyl]-2-(3-methylphenoxy)acetamide
TRAP	tartrate-resistant acid phosphatase
BMMs	bone marrow-derived macrophages
MNCs	multinucleated cells
M-CSF	macrophage colony-stimulating factor
RANK	receptor activator of nuclear factor-kappa Β
TRAF6	tumor necrosis factor receptor-associated factor 6
MAPKs	mitogen-activated protein kinases
ERK	extracellular signal-regulated
JNK	c-Jun N-terminus kinase
AP-1	activator protein 1
NFAT	nuclear factor of activated T-cells
Cts K	cathepsin K
MMP-9	matrix metalloprotease 9
DC-STAMP	dendritic cell-specific transmembrane protein
FACS	fluoresceinamine-labeled chondroitin sulfate
CaP	calcium phosphate
DMSO	dimethyl sulfoxide
OVX	ovariectomized
BV/TV	trabecular bone volume
Tb.N	trabecular number
Tb.Th	trabecular thickness
Tb.Sp	trabecular separation
CTX-1	type 1 collagen cross-linked C-terminal telopeptide
OPG	osteoprotegerin
