# Supplementary material for: N-[2-(4-Acetyl-1-Piperazinyl)Phenyl]-2-(3-Methylphenoxy)Acetamide (NAPMA) Inhibits Osteoclast Differentiation and Protects against Ovariectomy-Induced Osteoporosis
Source: Molecules. 2020 Oct 21;25(20):4855. doi: 10.3390/molecules25204855 (PMC7587973; doi:10.3390/molecules25204855)
Supplement: Supplementary file 1 [file molecules-25-04855-s001.pdf]

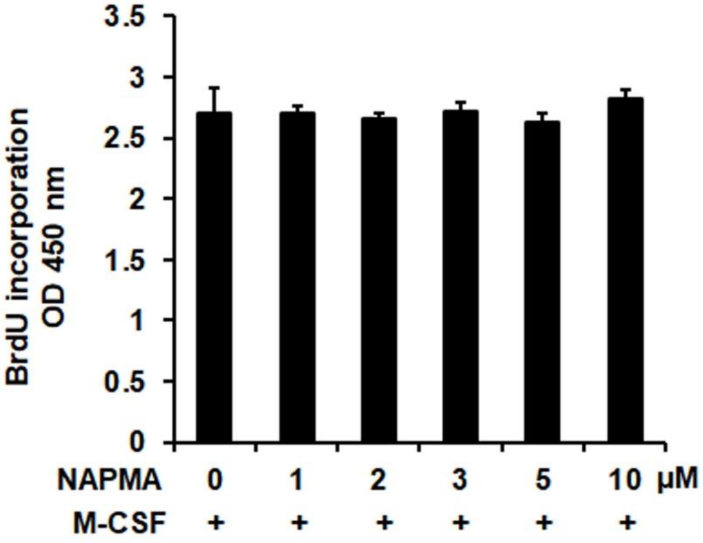

Figure S1. . NAPMA had no effect on the proliferation of BMMs. BMMs (1 × 10^4 cells/well) were cultured with M-CSF (30 ng/mL) and treated with different concentrations of NAPMA (0, 1, 2, 3, 5, and 10 μM) every day for 3 days. Cell proliferation was assessed using a BrdU Cell Proliferation Assay Kit. The data presented are the mean ± SD of four independent experiments.

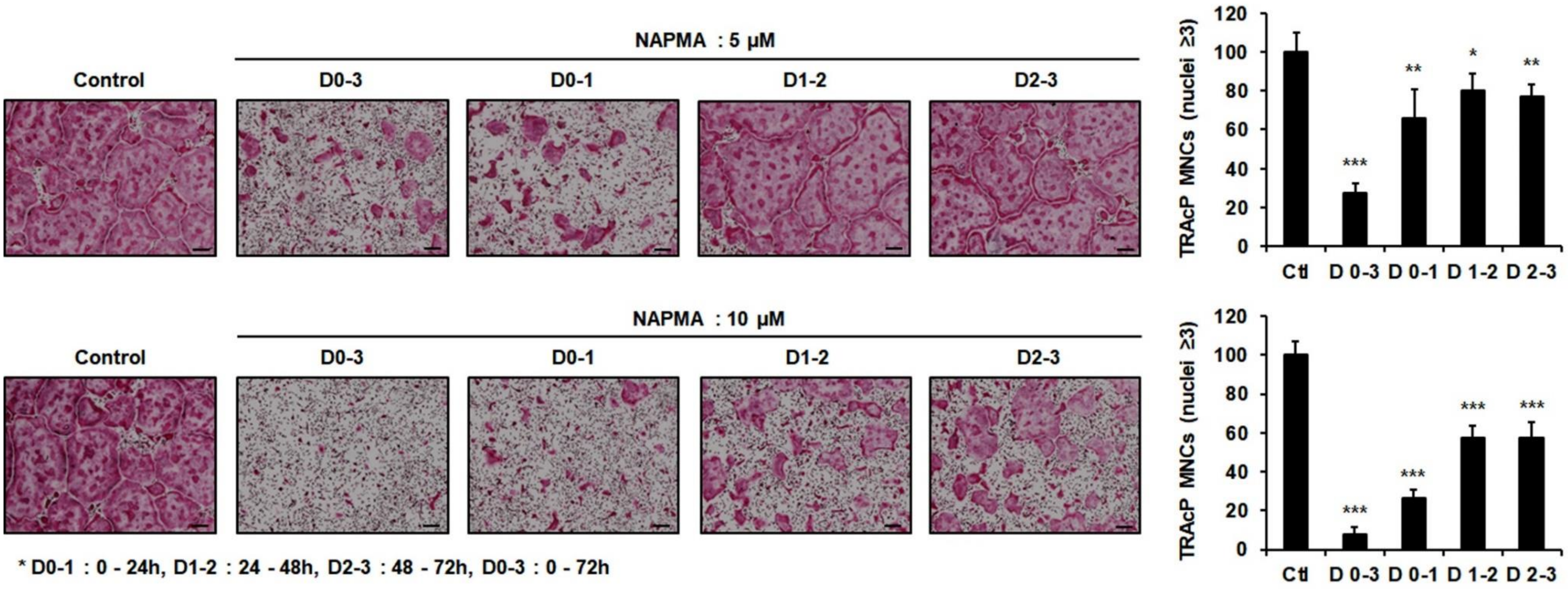

Figure S2. Effects of NAPMA on RANKL-induced signaling in BMMs. M-CSF (30 ng/mL)-treated BMMs were stimulated with RANKL (50 ng/mL) and treated with NAPMA (5 μM and 10 μM) at the indicated time points. The control samples were not treated with NAPMA. The data presented are the mean ± SD of four independent experiments. Scale bar = 200 μm.

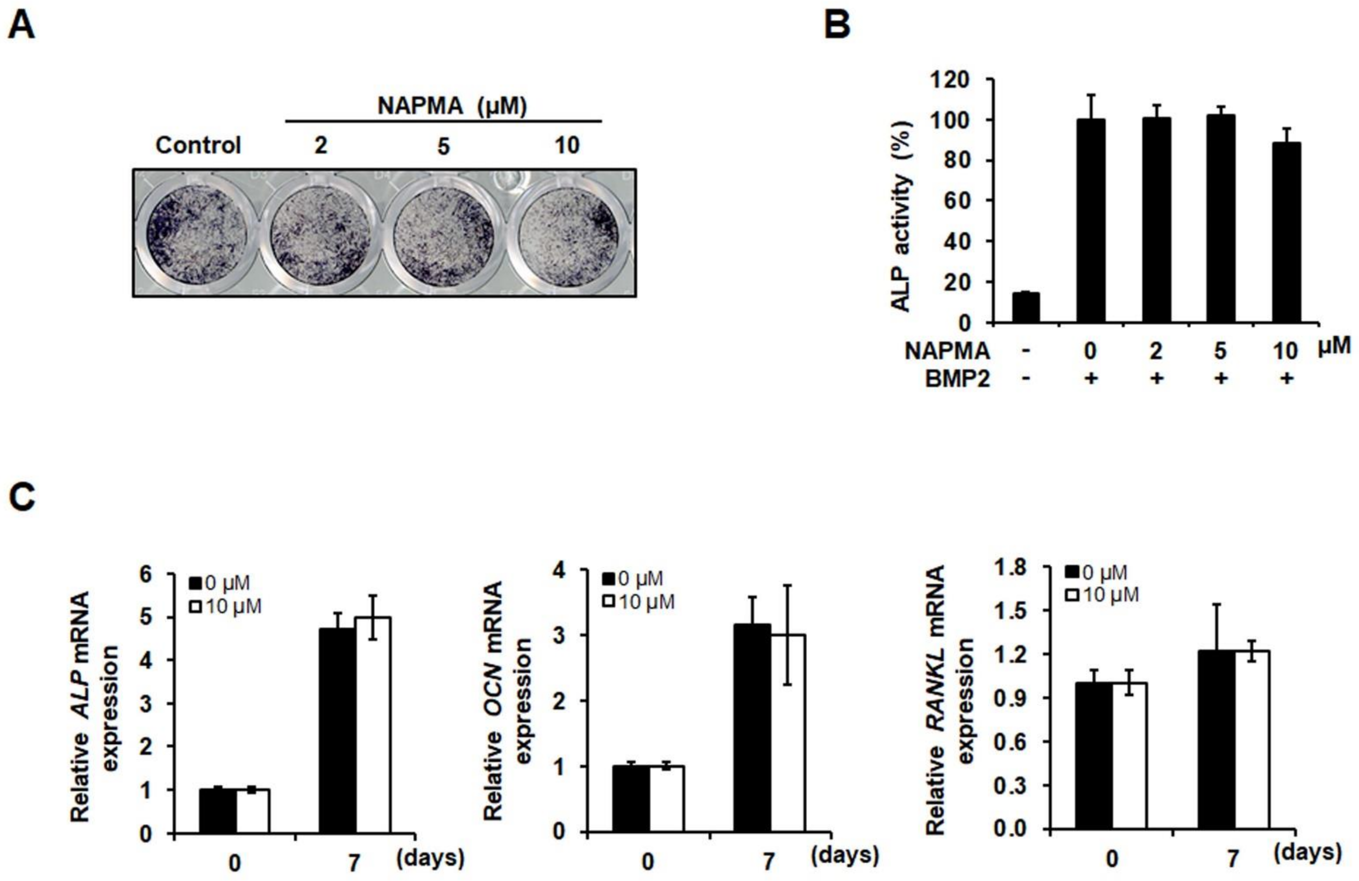

Figure S3. NAPMA had no effect on BMP-induced osteoblast differentiation. (A) Calvarial osteoblasts were cultured for 7 days and then stained for ALP, and (B) ALP activity was measured. (C) qPCR analysis of the expression of osteoblast-specific genes, ALP, RANKL, and osteocalcin (OCN) in calvarial osteoblasts treated with BMP-2 for 7 days in the presence of NAPMA. The data presented are the mean ± SD. All experiments were performed a minimum of three times.

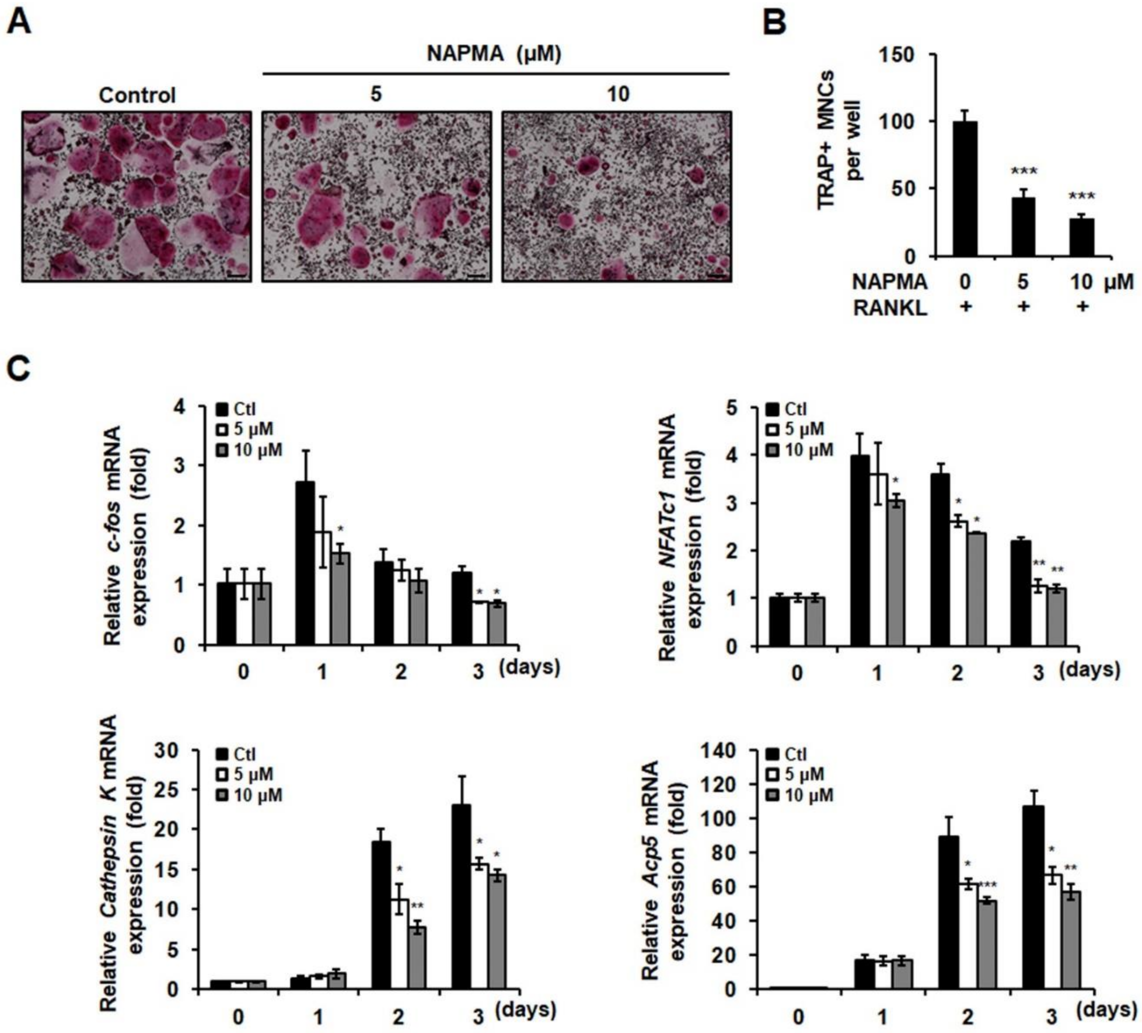

Figure S4. Effect of NAPMA on receptor activator of nuclear factor-kappa B (RANKL)-induced osteoclast differentiation in RAW264. 7 cells. (A) NAPMA inhibited osteoclast formation in a dose-dependent manner. RAW264.7 cells (1 × 105 cells/well) were treated with different concentrations of NAPMA for 3 days in the presence of RANKL (50 ng/mL). The cells were fixed and stained for the TRAP a ssay. (B) TRAP-positive multinucleated osteoclasts (≥3 nuclei) were counted. The data presented are the mean ± SD of four independe nt experiments. Scale bar = 200 μm. (C) qPCR analysis of the expression of osteoclast-specific genes, c-fos, NFATc1, cathepsin K (Cts K), Acp5 in RAW264.7 cells stimulated with RANKL for 3 days in the presence of NAPMA. The data presented are the mean ± SD of t hree independent experiments. *p<0.05, **p<0.01, and ***p<0.001 compared with the control group (treated with RANKL but without NAPMA).

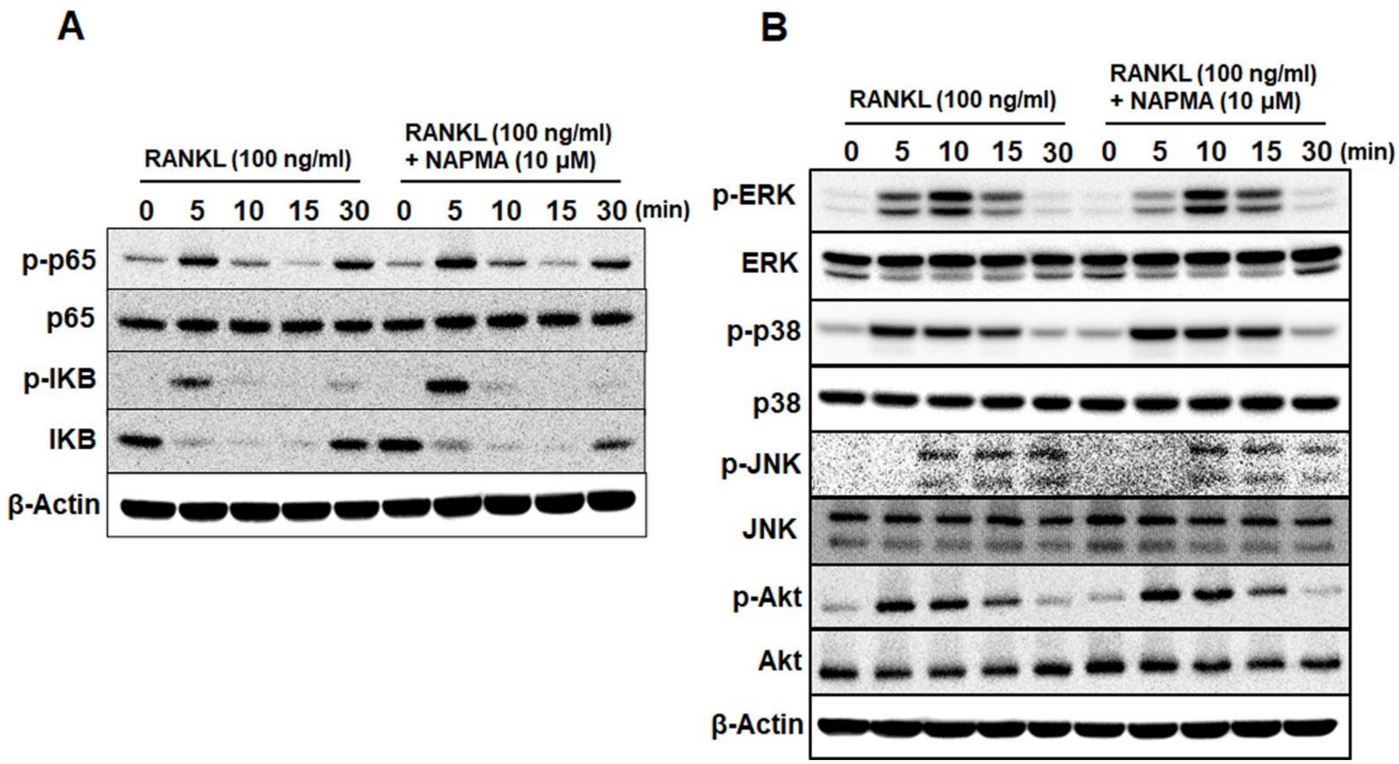

Figure S5**.** Effects of NAPMA on RANKL-induced signaling in BMMs. M-CSF-treated BMMs were pre-treated with 10 μM NAPMA or c ontrol (DMSO) for 1 h and then RANKL (100 ng/mL) was used to stimulate the cells at the indicated time points. The cells were analyzed using western blotting to detect (A) anti-phospho p65, IkB, (B) anti-phospho ERK, p38, JNK, and Akt signal proteins, as indicated.

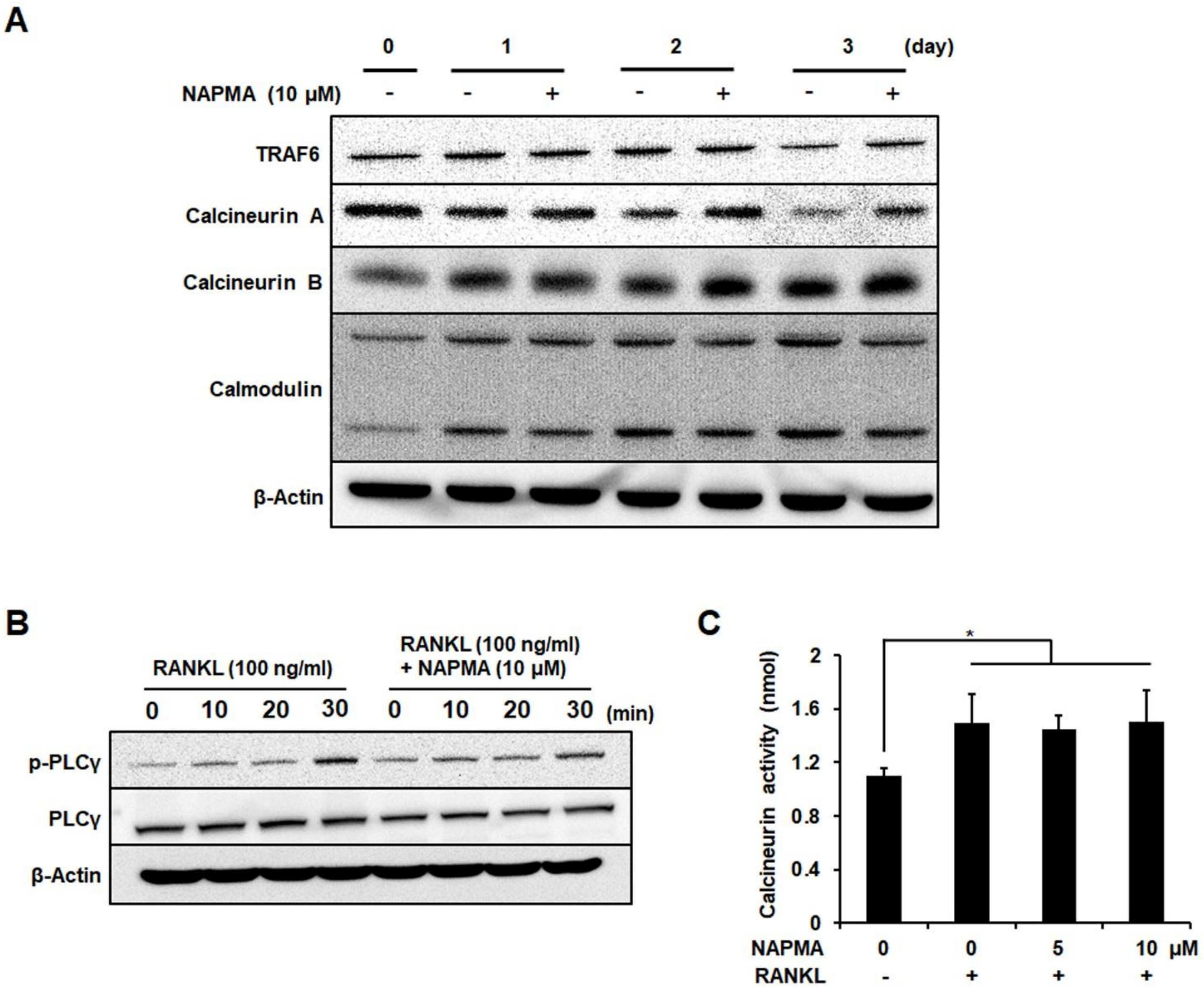

Figure S6. NAPMA does not inhibit the RANKL-induced expression of (A) TRAF6, calmodulin, calcineurin A or B proteins, and (B) anti-phospho PLCγ. BMMs were treated with or without NAPMA (10 μM) in the presence of RANKL (50 ng/mL) and M-CSF (30 ng/mL). Pro tein expression was examined using western blotting analysis for the indicated times. (C) Quantification of calcineurin activity in RAW264 .7 cells treated with RANKL in the presence or absence of NAPMA for 30 min. The data presented are the mean ± SD of three independent experiments. *$p<0.05$, **$p<0.01$, and ***$p<0.001$ compared with the control group (treated with RANKL but without NAPMA).

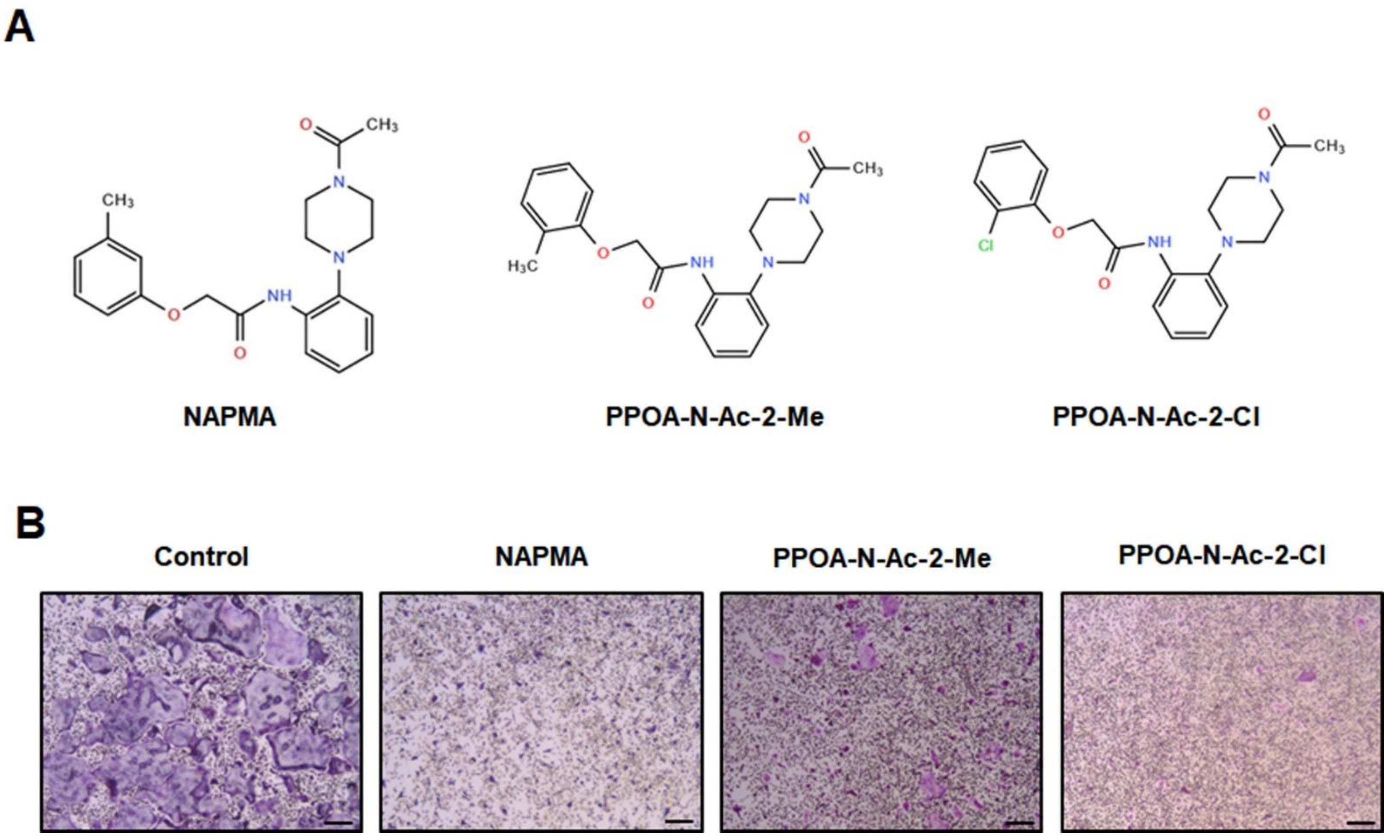

Figure S7**.** Effect of NAPMA, PPOA-N-Ac-2-Me, and PPOA-N-Ac-2-Cl on on RANKL-induced signaling in BMMs. (A) Structures of N APMA, PPOA-N-Ac-2-Me, and PPOA-N-Ac-2-Cl. (B) BMMs were differentiated into osteoclasts upon treatment with macrophage colon y-stimulating factor (M-CSF) (30 ng/mL) and receptor activator of nuclear factor-kappa B ligand (RANKL) (50 ng/mL) in the presence of each chemical (10 M each). The data presented are the mean ± SD of three independent experiments. Scale bar = 200 μm.

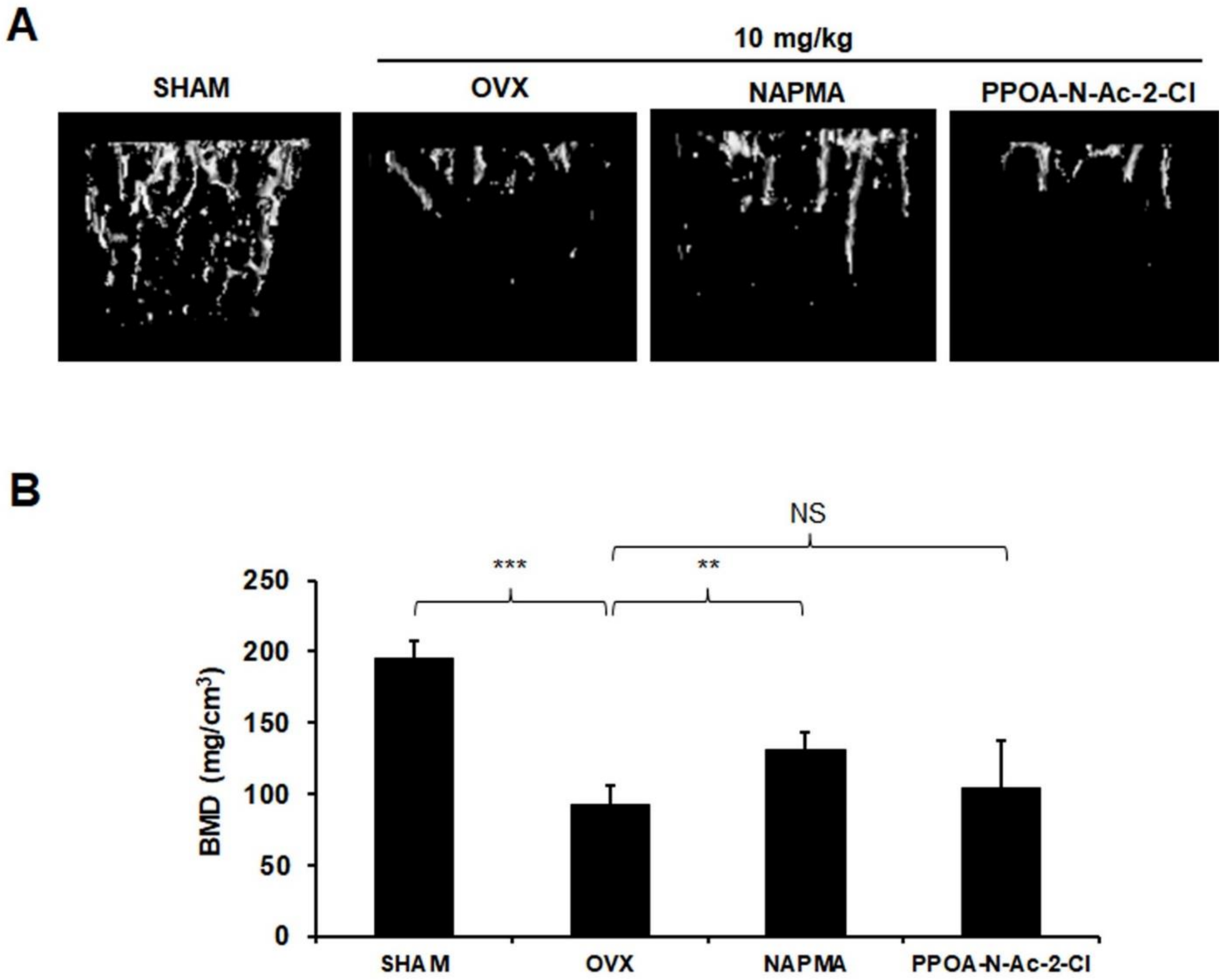

Figure S8**.** NAPMA treatment prevented OVX-induced bone loss. (A) OVX mice were euthanized after 6 weeks of NAPMA, PPOA-N-Ac-2-Me, or PPOA-N-Ac-2-Cl treatment. CT images of the distal femurs from the sham-operated group (SHAM), OVX group, and OVX tr eated with NAPMA, PPOA-N-Ac-2-Me, and PPOA-N-Ac-2-Cl (10 mg/kg/day) were obtained. (B) Bone densitometry was assessed in the SHAM, OVX model, and OVX mice treated with NAPMA, PPOA-N-Ac-2-Me, and PPOA-N-Ac-2-Cl (10 mg/kg/day) groups. The data ar e presented as the mean ± SD (n=6)..

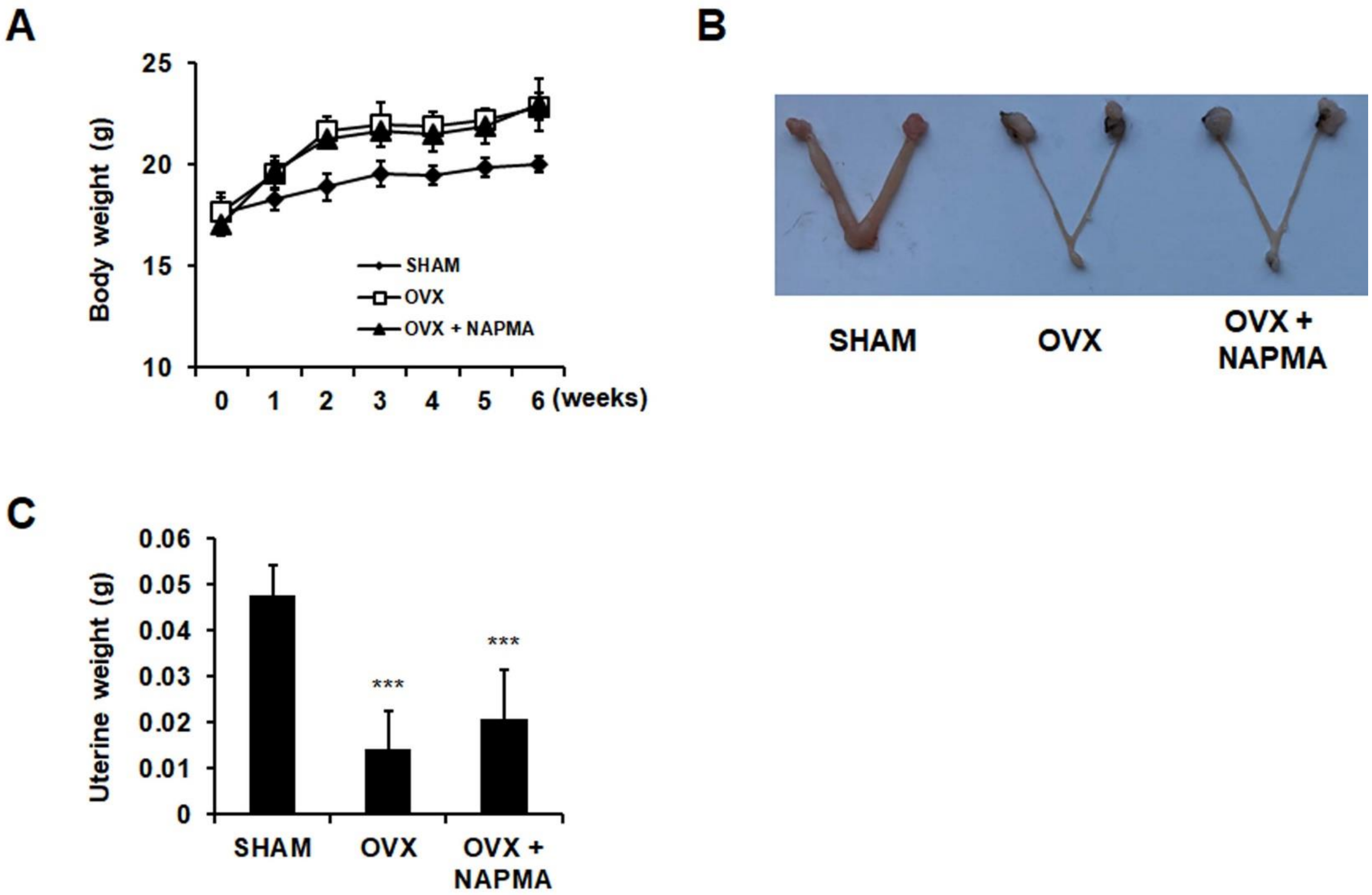

Figure S9**.** Validation of the success of ovariectomy. Six weeks after ovariectomy, all mice were euthanized. (A) The body weight of the mi ce was measured. NAPMA (10 mg/kg) had little effect on mouse body weight. The uteri were photographed (B) and weighed (C). The data presented are the mean ± SD (n=6).

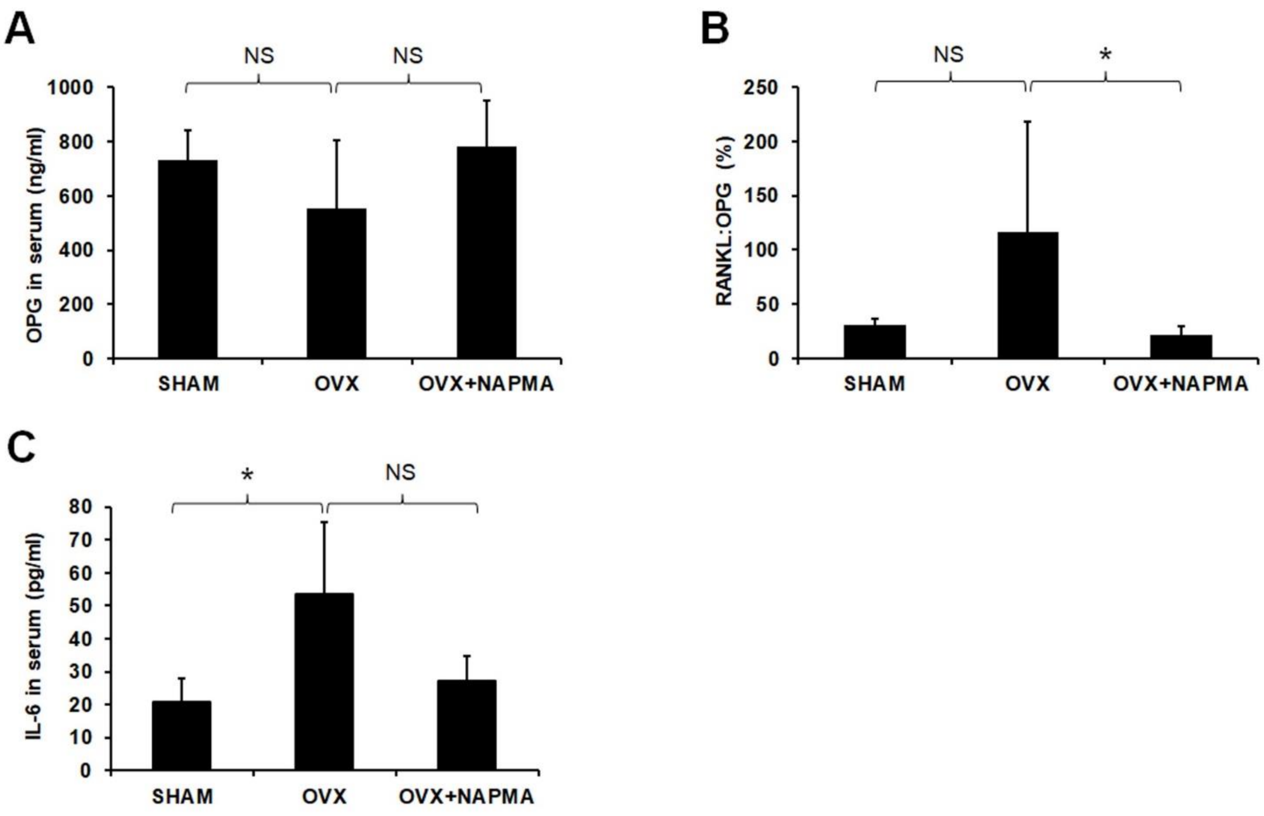

Figure S10**.** The serum level of OPG (A), the RANKL:OPG ratio (B), and IL-6 (C) of the animals was determined using ELISA. The data presented are the mean ± SD. n=6. *p<0.05; ***p<0.001.

Table S1. Primers used in this study.

Gene	Primer sequence (5'→3')	
GAPDH	Forward	TGTGTCCGTCGTGGATCTGA
	Reverse	GATGCCTGCTTCACCACCTT
ALP	Forward	AGTTCAGTGCGGTTCCAGACA
	Reverse	TGGCCTGGATCTCATCAGTATTT
RANKL	Forward	TGAAGACACACTACCTGACT
	Reverse	CCCACAATGTGTTGCAGTTC
OCN	Forward	GGACCTGTGCTGCCCTAAAG
	Reverse	AGAGAGGACAGGGAGGATCAAGT
